# MicroRNA Control of TGF-β Signaling

**DOI:** 10.3390/ijms19071901

**Published:** 2018-06-28

**Authors:** Hiroshi I. Suzuki

**Affiliations:** David H. Koch Institute for Integrative Cancer Research, Massachusetts Institute of Technology, Cambridge, MA 02139, USA; hisuzuki@mit.edu; Tel.: +1-617-253-6457

**Keywords:** miRNA, TGF-β, BMP, EMT, EndMT, tumor microenvironment

## Abstract

Transcriptional and post-transcriptional regulation shapes the transcriptome and proteome changes induced by various cellular signaling cascades. MicroRNAs (miRNAs) are small regulatory RNAs that are approximately 22 nucleotides long, which direct the post-transcriptional regulation of diverse target genes and control cell states. Transforming growth factor (TGF)-β family is a multifunctional cytokine family, which plays many regulatory roles in the development and pathogenesis of diverse diseases, including fibrotic disease, cardiovascular disease and cancer. Previous studies have shown that the TGF-β pathway includes the miRNA pathway as an important component of its downstream signaling cascades. Multiple studies of epithelial–mesenchymal transition (EMT)-related miRNAs have highlighted that miRNAs constitute the intrinsic bistable molecular switches of cell states by forming double negative feedback loops with EMT-inducing transcription factors. This may be important for understanding the reversibility of EMT at the single-cell level, the presence of distinct EMT transition states and the intra- and inter-tumor heterogeneity of cancer cell phenotypes. In the present review, I summarize the connection between TGF-β signaling and the miRNA pathway, placing particular emphasis on the regulation of miRNA expression by TGF-β signaling, the modulation of TGF-β signaling by miRNAs, the miRNA-mediated modulation of EMT and endothelial–mesenchymal transition as well as the crosstalk between miRNA and TGF-β pathways in the tumor microenvironment.

## 1. Introduction

The regulation of transcriptomes is a major consequence of various intracellular signaling cascades that are located downstream of growth factors and cytokines, which often represents their biological activities. These signaling cascades are usually directly linked to their corresponding transcription factors, which mediate the repression or activation of target genes. However, it is now clear that this transcriptional regulation is closely associated with epigenetic modification and post-transcriptional regulation, with the resulting transcriptomes being shaped by complex multilayered regulatory networks.

MicroRNAs (miRNAs) are small regulatory non-coding RNAs, which are approximately 22 nucleotides long. miRNAs form an RNA–protein complex with Argonaute proteins, recognize multiple target mRNAs via sequence complementarity and repress target RNAs, thereby serving as the major players in the post-transcriptional gene regulation of diverse species [1]. In humans, many conserved miRNAs display preferentially conserved interactions with hundreds of target mRNAs and thus, they exhibit diverse effects on transcriptomes [2]. To date, hundreds of distinct miRNAs have been identified in humans using stringent criteria, while thousands of human genes have been shown to be miRNA targets [2,3,4]. Reflecting the diversity of their targets and their involvement in various biological pathways, miRNAs have been shown to regulate multiple cellular pathways and to play important roles in multiple aspects of development, physiology and disease pathogenesis [5]. For example, multiple loss-of-function studies have shown that depletion of miRNA genes leads to developmental, physiological and/or behavioral abnormalities [1,6]. Furthermore, miRNAs have also been implicated in many biological characteristics of cancer (i.e., hallmarks of cancer) [7].

Significant evidence has indicated that miRNAs are involved in the post-transcriptional gene regulation in various conserved cellular signaling cascades [8,9]. These signaling cascades include the transforming growth factor (TGF)-β, Notch, Hedgehog and mitogen-activated protein kinase (MAPK) pathways. The downstream targets of these pathways include multiple downstream miRNAs, which provide positive or negative feedback to downstream signal mediators or transcription factors [10,11].

The TGF-β family is a multifunctional cytokine family that regulates multiple cellular functions, including cell growth, differentiation, adhesion, migration and death [12,13]. TGF-β signaling has many regulatory roles in development, with alterations in this signaling pathway having been associated with the pathogenesis of various diseases, including fibrotic disease, cardiovascular disease and cancer [14,15,16,17]. Previous studies have shown that the TGF-β signaling pathway embraces the miRNA pathway as an important component of its downstream signaling cascades [18,19,20,21,22,23,24]. In the present review, I summarize the connection between TGF-β signaling and miRNAs with a particular focus on: (1) regulation of miRNA expression by TGF-β signaling; (2) modulation of TGF-β signaling by miRNAs; (3) miRNA-mediated regulation of cell state transitions, such as epithelial–mesenchymal transition (EMT) and endothelial–mesenchymal transition (EndMT); and (4) crosstalk between miRNA and TGF-β pathways in cancer.

## 2. Biogenesis of miRNAs

In mammals, many miRNAs are first generated as a part of a longer primary miRNA transcript (pri-miRNA) transcribed by RNA polymerase II [25]. A hairpin structure of pri-miRNAs is cleaved by the microprocessor complex, which is composed of RNase III Drosha and its cofactor DGCR8 in the nucleus. The microprocessor is a heterotrimeric complex with one Drosha and two DGCR8 molecules that generates stem-loop structured RNAs, which are termed pre-miRNAs, by cleaving the stem region of the hairpin structure of pri-miRNAs [26]. Several proteins have been shown to be associated with the microprocessor complex and regulate its processing activity [27,28,29]. Pre-miRNAs are transported to the cytoplasm by Exportin 5 and RAN-GTP, where they undergo further cleavage by the RNase III enzyme Dicer [30,31]. Dicer processes the pre-miRNA to a double-stranded ~22-nucleotide product that is known as the miRNA duplex, which consists of the miRNA guide strand and passenger strand. In mammals, Dicer associates with the partner proteins HIV transactivation response element (TAR)-binding protein (TRBP) and protein activator of the interferon-induced protein kinase (PACT) [32].

The miRNA duplex is sequentially loaded onto Argonaute proteins (Ago1–Ago4 in mammals) with the help of HSP70/90 chaperone machinery [33]. Finally, one strand, whose 5′-end is captured by the MID domain of Argonaute, is retained in the complex, while the other strand protrudes from Argonaute to form the mature RNA-induced silencing complex. By convention, the more abundant strand is referred to as the miRNA or guide strand, whereas the less abundant strand is known as the miRNA* or the passenger strand. The selection of which strand serves as the guide strand is an asymmetric process. In mammals, this asymmetric selection is directed by the Argonaute proteins [34]. Mammalian Argonaute proteins select strands with 5′-uridine/adenosine and thermodynamically unstable 5′-ends with its two sensor regions, which come into contact with the 5′-nucleobase and 5′-phosphate(s) of prospective guide strands, respectively. Thus, the asymmetry of mammalian miRNAs shows unique patterns that reflect two independent molecular rules for the 5′-end nucleotide identity and thermodynamic stability of each miRNA duplex [34]. Many miRNA duplexes yield a single dominant mature miRNA but often provide both strands as mature miRNAs [3].

Thus, the biogenesis of miRNAs consists of multiple steps, including transcription, processing by Drosha and Dicer and Argonaute loading. This is further influenced by modification by RNA editing, RNA methylation, uridylation, adenylation and RNA decay. The expression of each miRNA species can be regulated at each step [29,35,36]. In fact, the post-transcriptional regulation of miRNA biogenesis has been well studied, while many RNA binding proteins (RBPs) and factors have been reported as post-transcriptional or co-transcriptional modulators of miRNA biogenesis [35,36,37,38,39,40].

## 3. Function and Expression of miRNAs

In the recent version of miRBase (miRBase v21), 1193 mouse and 1881 human miRNA gene annotations have been registered [41]. Using stringent criteria, the numbers of confidently identified canonical miRNA genes are 475 and 519 in mice and humans, respectively [1,3,4].

In canonical miRNA-based targeting, the recognition of target mRNAs by miRNAs is mediated primarily through sequence complementarity between the miRNA seed (miRNA nucleotides 2–7) and its target site in the 3′-untranslated region (UTR) of target mRNAs [42]. Due to dependence on short seed sequences for target recognition, each miRNA can potentially target hundreds of genes and the emergence of miRNA target sites in 3′-UTRs has influenced 3′-UTR evolution [43,44]. Accordingly, many conserved miRNAs have been shown to exhibit preferentially conserved interactions with hundreds of target mRNAs [2]. The evolution of 3′-UTRs has been associated with the evolutionary conservation of tissue-specific expression patterns of tissue-specific miRNAs [43,44].

In animals, target repression through partial complementarity involves two modes of target repression, mRNA decay and translational inhibition although they do not target degradation by Argonaute-dependent endonucleolytic cleavage triggered by extensive complementarity [1]. Canonical miRNA-mediated target repression largely depends on the adapter proteins of Argonaute, TNRC6A/B/C. These adapter proteins induce shortening of the poly(A) sequence, subsequent mRNA destabilization and translational repression. It has been reported that mRNA destabilization rather than translation repression dominates target repression by mammalian miRNAs in post-embryonic cells, except for in the early embryo [45,46,47].

Genome-wide expression profiling, including miRNA microarray and small RNA sequencing analyses, has identified hundreds of miRNAs at detectable levels in a single cell type. However, only a small number of miRNAs predominate the total miRNA expression in multiple cell types [48,49]. This is also the case for miRNA function, while a few abundant miRNAs mediate the marked target repression at the population level [50,51]. Importantly, loss-of-function studies have highlighted the importance of such tissue-specific abundant miRNAs in the development and pathogenesis of disease [6]. We have recently shown that these quantitative features of miRNA-mediated gene regulation can be explained by an association between super-enhancers and miRNA genes [52]. Super-enhancers are the major drivers of tissue-specific highly biased miRNA expression and function. Moreover, super-enhancers can also explain alterations of the multiple miRNAs that are involved in tumor pathogenesis [52].

## 4. Overview of TGF-β Signaling

The TGF-β family consists of three TGF-β isoforms, activins, nodal, bone morphogenetic proteins (BMPs) and growth and differentiation factors [12,13]. These members regulate multiple cellular functions, including cell growth, differentiation, adhesion, migration and death in a context-dependent and cell type-specific manner [13,53,54].

TGF-β family ligands bind to two different types of serine-threonine kinase receptors, which are the type II and type I receptors (TβR-II and TβR-I for TGF-β). Upon ligand binding, specific tetrameric type II/type I receptor complexes are activated to transduce downstream signals. Type I receptors phosphorylate the receptor-regulated Smads (R-Smads). Typically, TGF-β isoforms and activins trigger the phosphorylation of Smad2 and Smad3 (activin/TGF-β-specific R-Smads), while BMPs trigger the phosphorylation of Smad1, Smad5 and Smad8 (BMP-specific R-Smads). Thus, TGF-β family members can be largely divided into two groups according to the downstream R-Smads [12]. Subsequently, R-Smads form complexes with a common-partner Smad (co-Smad). Smad4 is the only co-Smad in mammals that is shared with the TGF-β family signaling pathways. The R-Smad/co-Smad complexes accumulate in the nucleus and regulate the transcription of various target genes together with a range of transcription factors and transcriptional modulators [53,54]. The inhibitory Smads (I-Smads), which are namely Smad6 and Smad7, can inhibit R-Smad activation and overall signal strength [55]. Additionally, TGF-β activates several Smad-independent signal transduction pathways, including the phosphatidylinositol 3 kinase (PI3K)-Akt and MAPK pathways. These are referred to as non-Smad signaling pathways [56,57].

TGF-β signaling has been shown to play multiple important roles in cancer progression [15,16,17,54]. These can be largely categorized into four distinct processes: (1) growth modulation, particularly escape from growth inhibition by TGF-β at the early stage of cancer initiation; (2) enhanced synthesis of the extracellular matrix and fibrosis by TGF-β in the tumor microenvironment; (3) promotion of EMT and/or metastasis by TGF-β; and (4) immune suppression by TGF-β in the tumor microenvironment. TGF-β has been proposed to function both as a tumor suppressor and a tumor promoter, depending on the cancer type and the stage of cancer progression [15]. It is thought that TGF-β serves as a tumor suppressor through the inhibition of cell growth in the early stages of carcinogenesis. On the other hand, it has been suggested that TGF-β promotes tumor progression in the advanced stages of cancer by enhancing cancer cell migration, tissue fibrosis and/or immune suppression [58]. To date, it has been suggested that multiple miRNAs contribute to these processes in relation to TGF-β signaling [18,19,20,21,22,23,24].

## 5. Regulation of miRNA Expression and Biogenesis by TGF-β Signaling

**Figure 1:**
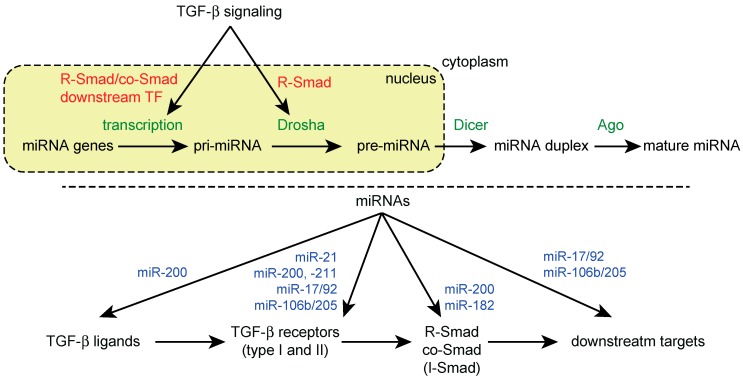
Reciprocal crosstalk between TGF-β signaling and miRNA machinery. (**Top**) Regulation of miRNA biogenesis by TGF-β signaling; (**Bottom**) Regulation of TGF-β signaling components by miRNAs. Examples of miRNAs targeting TGF-β signaling components are shown.

Multiple studies have shown that treatment with TGF-β family ligands induces changes in the expression levels of multiple miRNAs in different cell types [18,19,20,21,22,23,24]. TGF-β signaling has been shown to modulate miRNA expression at both the transcriptional and post-transcriptional levels (Figure 1, top). In particular, early studies have shown that R-Smads directly influence miRNA biogenesis by modulating Drosha-mediated pri-miRNA processing, which demonstrates an unexpected transcription-independent function of Smads [59,60].

TGF-β and BMP signaling have been shown to increase the expression of mature miR-21 in human vascular smooth muscle and breast cancer cells [59]. This increase in miR-21 expression is accompanied by the upregulation of pre-miR-21 but not pri-miR-21. Treatment with TGF-β and BMP4 has been shown to induce an association between R-Smads and the cofactor of the microprocessor p68 on pri-miR-21, which is thought to facilitate pri-miR processing. In contrast to the canonical Smad pathway, the co-Smad Smad4 is not required for this ligand-dependent pri-miRNA processing [59]. The pre-miRNAs of TGF-β/BMP-regulated miRNAs contain a conserved sequence, CAGAC, which is similar to the Smad binding element found in the promoters of TGF-β/BMP-regulated genes [60]. Smads directly interact with this RNA-Smad binding element (R-SBE) through their N-terminal MH1 domain, while TGF-β/BMP-induced pri-miRNA processing depends on R-SBE. R-SBE has been identified in several other TGF-β/BMP-induced miRNAs, including miR-105, miR-199a, miR-215, miR-421 and miR-529 [60]. Additionally, it has been suggested that a Smad4-independent mechanism can mediate TGF-β-induced miR-181a upregulation [61], although both Smad4-independent post-transcriptional and Smad4-dependent transcriptional mechanisms are thought to be important in the TGF-β-dependent induction of the miR-181 family [62,63]. This type of post-transcriptional regulation of miRNA biogenesis has also been demonstrated for other transcriptional regulators, including p53 and Nanog [64,65]. These observations suggest that microprocessor cofactors, such as p68 and p72, as well as other RBPs serve as the molecular interfaces for integrating intracellular signaling pathways into the miRNA pathway [29,37].

TGF-β/BMP-regulated miRNAs can be extended to other miRNAs in different cell types. The miRNAs upregulated by TGF-β signaling include miR-21, the miR-181 family, miR-10b, the miR-17/92 cluster, miR-155, miR-192, the miR-23/24/27 cluster, miR-216/217, miR-494 and miR-182 [18,24,66,67,68,69,70,71,72,73]. The miRNAs downregulated by TGF-β signaling include the miR-200 family, miR-203, let-7, miR-34a and miR-584 [18,24,74,75,76,77]. In some cases, transcriptional mechanisms have been investigated. In normal murine mammary gland (NMuMG) epithelial cells, TGF-β activates the miR-155 promoter and induces miR-155 expression through Smad4 [68]. miR-494 is also induced by TGF-β at the pri-miRNA level in a Smad4-dependent manner, while miR-494 shows reduced expression in pancreatic cancer cells with a loss of Smad4 [72].

Additionally, the downstream transcription factors of TGF-β signaling have been shown to regulate multiple miRNAs at the transcriptional level. This is easily recognized with EMT-related miRNAs. As discussed later, several miRNAs, including the miR-200 family, miR-203 and miR-216/217, are suppressed by EMT-related transcriptional regulators, such as ZEB1 (also known as δEF1), ZEB2 (also known as SIP1), SNAIL, SLUG and E-box-dependent mechanisms, which are located downstream of TGF-β signaling [71,74,78,79,80,81].

## 6. Regulation of TGF-β Signaling by miRNAs

TGF-β signaling and miRNA pathways have been shown to exhibit reciprocal crosstalk. Consistent with the diversity of miRNA target genes, the computational prediction of miRNA targets suggests that multiple components of the TGF-β signaling pathway are targeted by multiple miRNAs. In fact, several miRNAs have been experimentally validated to be modulators of TGF-β signaling at multiple levels by targeting ligands, receptors, R-Smad, co-Smad, I-Smad and non-Smad pathway components as well as downstream targets of TGF-β signaling (Figure 1, bottom) [18,20,24].

Many miRNAs targeting TGF-β receptors, especially the type II receptor, have been identified in various cancer types. These miRNAs, which include miR-21, the miR-17/92 cluster, miR-106b, miR-211 and miR-590 [82,83,84,85,86,87], are frequently oncogenic [20,24]. These miRNAs promote proliferation, invasion or resistance to chemotherapeutic drugs in different cancer cell types.

Intriguingly, some TGF-β-regulated miRNAs target TGF-β signaling components, thus comprising the feedback regulation of TGF-β signaling. The miR-200 family, which consists of TGF-β-downregulated miRNAs, targets TGF-β, TβR-I and Smad2 [88,89]. Thus, the downregulation of the miR-200 family by TGF-β enhances TGF-β signaling and induction of EMT. miR-182, a TGF-β-inducible miRNA, targets Smad7 and thus, modulates a negative feedback loop of TGF-β signaling as Smad7 is also induced by TGF-β [90]. As a result, miR-182 potentiates the TGF-β-induced EMT and metastasis of cancer cells.

The miRNAs also regulate TGF-β signaling by convergently suppressing a range of downstream TGF-β target genes. The miR-106b/205 cluster that is composed of E2F1-regulated miRNAs targets the TGF-β downstream effectors, p21^Waf1/Cip1^ and Bim, and disrupt TGF-β-dependent cell cycle arrest and apoptosis in gastric cancer [91]. Additionally, the miR-17/92 cluster, which targets multiple TGF-β signaling components, such as TβR-I, Smad2 and Smad4, suppresses the TGF-β-responsive genes p21^Waf1/Cip1^ and Bim. This ultimately inhibits the effects of TGF-β signaling [84,92,93].

## 7. Control of Epithelial Identity and Epithelial–Mesenchymal Transition (EMT) by the miR-200 Family

The dynamic and reversible regulation of cell identity is important for the organization of complex multicellular systems and for adaptation under diverse physiological and pathological conditions. EMT, which converts the epithelial cells into mesenchymal cells, is a well-known example of such cellular plasticity, with the regulation of EMT being involved in the development and pathogenesis of cancer [94]. TGF-β is known to induce EMT. Previous studies have identified multiple miRNAs that are involved in EMT and have shown that both downstream transcription factors and miRNAs orchestrate the conserved TGF-β-inducible EMT programs. In this section, I focus on the miR-200 family as its role in EMT has been extensively studied [78,79,80,81].

The miR-200 family consists of five miRNAs with similar seed sequences: miR-200b, miR-200c and miR-429 with the seed sequence AAUACU and miR-200a and miR-141 with the seed sequence AACACU, which differ by a single nucleotide [95,96,97]. These miRNAs are encoded in two transcriptional clusters, which are namely the miR-200b/200a/429 and miR-200c/141 clusters. Consistent with their roles in epithelial identity, these miRNA clusters are closely associated with super-enhancers found in digestive organs [52].

**Figure 2:**
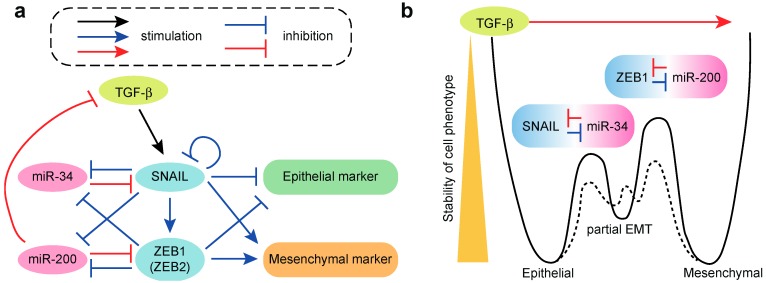
Roles of miRNAs in TGF-β-induced epithelial–mesenchymal transition (EMT). (**a**) EMT circuits containing a SNAIL-miR-34 loop and ZEB1-miR-200 loop; (**b**) A relationship between two miRNA-transcription factor (TF) modules and three EMT states. Solid line indicates the cell state transitions among epithelial, partial EMT and mesenchymal phenotypes, which are controlled by two miRNA-TF molecular switches. Dotted line suggests the alternative transition states generated by additional molecular switches.

Members of the miR-200 family are markedly downregulated following TGF-β treatment in multiple EMT models using various cell lines, including Madin Darby canine kidney (MDCK) epithelial cells, NMuMG epithelial cells and MCF10A mammary epithelial cells [78,80,98]. Forced expression of the miR-200 family is sufficient for preventing the TGF-β-induced EMT in MDCK cells and inducing mesenchymal–epithelial transition (MET) in mesenchymal cells. This suggests that the miR-200 family acts as an enforcer of epithelial identity [78,79,80,81]. The promoter regions of the miR-200b/200a/429 and miR-200c/141 clusters are repressed by the TGF-β-induced transcriptional repressors ZEB1 and ZEB2 during EMT through ZEB-type E-box elements [81,99]. Furthermore, prolonged exposure to TGF-β can induce reversible DNA methylation of miR-200 family promoters [88]. Conversely, the miR-200 family targets the 3′-UTRs of ZEB1/2 [78,79,80,81]. Thus, ZEB1/2 and the miR-200 family form a double negative feedback loop, which controls epithelial identity (Figure 2a). Additionally, the maintenance of a stable mesenchymal phenotype requires TGF-β autocrine signaling, which is supported by the release of miR-200-mediated repression of TGF-β ligand production in MDCK cells. This suggests a more complex feedback network involving TGF-β, ZEB1/2 and the miR-200 family [88]. The clinical importance of this feedback loop is supported by a positive correlation between ZEB1/2 and TGF-β; by negative correlations between miR-200 and TGF-β and between miR-200 and ZEB1/2 in breast cancer [78,88]; and by an association between high miR-200 expression and better clinical outcomes in multiple cancer types [100].

An unbiased investigation of miR-200 targets using Ago2 HITS-CLIP (high-throughput sequencing after cross-linked immunoprecipitation) has identified hundreds of miR-200a and miR-200b targets. This study has revealed an important effect of miR-200 on actin cytoskeleton dynamics [101]. Thus, the miR-200 family is important for maintaining epithelial identity and prevents cell migration by regulating a core EMT transcriptional program and multiple regulators of Rho-ROCK signaling, focal adhesion, matrix metalloproteinase activity and invadopodia formation. In addition to their regulation of cell motility and metastasis, the miR-200 family also plays important roles in regulating drug resistance in cancer therapy [95,96,97]. The crosstalk between miR-200 and ZEB1/2 has also been demonstrated in stem cell biology. The induction of the miR-200 family and MET occurs in the initial phase of the reprogramming of somatic cells to induced pluripotent stem (iPS) cells, facilitating their generation [102,103].

## 8. miRNA Regulation and miRNA-Mediated Bistable Switches of the TGF-β-Mediated EMT

Similar to the miR-200 family, additional miRNAs have also been shown to be associated with EMT. miR-205 is downregulated after TGF-β treatment and suppresses ZEB1/2 like the miR-200 family in MDCK cells [78]. ZEB1 has also been shown to repress several other miRNAs, such as miR-203 and miR-183, which cooperatively repress stem cell factors and inhibit the stemness property in cancer cells and mouse embryonic stem cells [104]. Additionally, several TGF-β-upregulated miRNAs, such as miR-181, miR-155 and miR-10b, promote EMT [61,66,68]. Other EMT-related miRNAs include miR-30, the miR-34 family and miR-223 [89,105,106,107].

The members of the miR-34 family, which are namely miR-34a and miR-34b/c, are suppressed by the EMT-inducing transcription factor SNAIL and conversely, the miR-34 family targets the 3′-UTR of SNAIL [105,106]. Thus, SNAIL and the miR-34 family comprise another double negative feedback loop controlling EMT, which is similar to the ZEB1/2-miR-200 feedback loop (Figure 2a). Detailed quantitative measurement of the dynamics of the TGF-β-induced EMT in MCF10A cells at the population and single-cell levels suggests that the two transcription factor-miRNA double negative feedback loops (i.e., the SNAIL-miR-34 and ZEB1-miR-200 loops) form bistable cell fate switches in the epithelial phenotype, partial EMT phenotype and mesenchymal phenotype (Figure 2a) [98,108]. These three phenotypes are characterized by high and low; medium and medium; and low and high abundance of E-cadherin and vimentin, respectively. The time course measurements of the TGF-β-induced EMT have suggested a two-step process among the three cell phenotypes (Figure 2b). In the first step, the epithelial cells transit to the partial EMT state and the SNAIL-miR-34 bistable switch is activated, which leads to reduced expression of E-cadherin and increased expression of vimentin. In the second step, the cells transit from the partial EMT state to the mesenchymal stat, and the ZEB1-miR-200 bistable switch is activated, which leads to reduced expression of E-cadherin and robust induction of vimentin expression [98]. In MCF-10A cells, it appears that the transition from epithelial to partial EMT state is reversible, whereas the transition from partial EMT to the mesenchymal state is irreversible, especially at high concentrations of TGF-β. In summary, in MCF10A cells, the TGF-β-induced EMT proceeds through the stepwise activation of the SNAIL-miR-34 and ZEB1-miR-200 modules in the initial and later phases, respectively [98,108]. This model will be important in furthering our understanding of the reversibility of EMT at the single-cell level and in interpreting the heterogeneity in EMT phenotypes, including partial EMT, in vivo and in clinical samples.

## 9. Regulation of TGF-β-Induced EndMT by miRNAs

TGF-β also plays important roles in the plasticity of other cell types. TGF-β is a major inducer of EndMT, which is a process that is similar to EMT. This process converts differentiated endothelial cells into mesenchymal cells [109]. EndMT is observed in cardiac development and diverse pathological conditions, including tissue fibrosis, cavernous malformations, vein stenosis and graft remodeling and cancer [110,111,112,113,114]. Several reports have recently described changes in the expression levels of multiple miRNAs during EndMT and the regulatory roles of several miRNAs in EndMT in different endothelial cells [115,116].

In the embryonic heart, miR-23 is necessary for restricting endocardial cushion formation by inhibiting TGF-β-induced EndMT and extracellular hyaluronic acid production [117]. miR-126, which is specifically expressed in endothelial cells and closely associated with super-enhancers found in human umbilical endothelial cells [52], has been reported to inhibit the TGF-β-induced EndMT through modulation of the PIK3R2-PI3K/Akt signaling pathway [118]. This suggests an analogous role of miRNAs in maintaining endothelial cell identity in addition to an essential role of the miR-200 family in epithelial cells. In a recent report, metastasis-associated lung adenocarcinoma transcript 1 (MALAT1) long non-coding RNA (lncRNA) was reported to be induced by TGF-β, which modulates the TGF-β-induced EndMT by suppressing miR-145. This suggests a complex network between lncRNAs and small RNAs [119]. Other reports have described the regulatory roles of several miRNAs, such as miR-21, miR-302c, miR-18a and miR-20a, in EndMT [120,121,122,123].

We have also reported that several miRNAs modulate EndMT in MS-1 mouse pancreatic microvascular endothelial cells [124,125]. Constitutively active miR-31 positively regulates the induction of mesenchymal markers during the TGF-β-induced EndMT in MS-1 endothelial cells without concomitant effects on the induction of TGF-β target genes and downregulation of endothelial markers. Additionally, miR-31 potentiates the induction of various chemokines and cytokines, including CCL17, CX3CL1, CXCL16, interleukin (IL)-6 and Angptl2, which has been designated as the EndMT-associated secretory phenotype (EndMT-SP). TGF-β induces alternative polyadenylation-mediated exclusion of the internal poly(A) sequence in the 3′-UTR of Stk40, which is a negative regulator of the NF-κB pathway. This enhances miR-31-dependent Stk40 suppression without concomitant miR-31 induction [124]. We also identified miR-27b, a member of the miR-23/24/27 cluster, as a TGF-β-inducible miRNA in MS-1 cells and observed that inhibition of miR-27 suppressed the induction of mesenchymal markers by TGF-β treatment [125].

## 10. TGF-β-Related miRNAs and the Tumor Microenvironment

**Figure 3:**
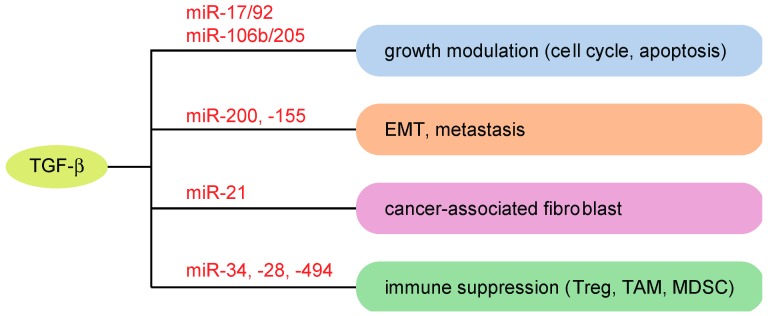
Roles of TGF-β-related miRNAs in cancer progression. Examples of miRNAs modulating TGF-β-associated cancer properties are shown. Treg: regulatory T cells; TAM: tumor-associated macrophages; MDSC: myeloid-derived suppressor cells.

miRNAs have diverse functions in cancer progression and are linked to wide aspects of cancer hallmarks. We have previously shown that the alterations of super-enhancers are observed for various cancer-related miRNAs associated with cancer hallmark traits [52]. Previous studies have demonstrated multifaceted contributions of multiple miRNAs to the roles of TGF-β signaling in cancer progression, including modulation of growth, EMT and metastasis, which have been discussed earlier (Figure 3) [18,19,20,21,22,23,24]. Additionally, recent advances in miRNA research have revealed that miRNAs regulate not only the properties of cancer cells but also the tumor microenvironment to facilitate tumor metastasis, resistance to cancer therapy and immune suppression [126]. This is also the case for TGF-β-related miRNAs. In this section, I summarize the non-cell-autonomous functions of TGF-β-related miRNAs and their roles in the cancer microenvironment (Figure 3).

Alterations of miRNAs in cancer cells can foster a favorable microenvironment for cancer cells through the altered production of factors secreted from cancer cells. In hepatocellular carcinoma (HCC), increased TGF-β signaling, which is associated with persistent hepatitis B virus (HBV) infection, results in suppression of miR-34a and subsequently enhances the production of the chemokine CCL22, which promotes the recruitment of regulatory T cells [127]. In HBV-positive HCC and portal vein tumor thrombus, miR-34a expression levels are inversely correlated with the expression levels of CCL22 and FoxP3, which strengthens the importance of the TGF-β-miR-34a-CCL22 axis [127]. Additionally, in HCC, TGF-β secreted from tumor-associated macrophages (TAMs) suppresses miR-28 in cancer cells [128]. Decreased expression of miR-28 results in increased secretion of its target IL-34 and enhances the infiltration of TAMs. This miRNA-mediated feedback loop between cancer cells and TAMs modulates HCC metastasis.

TGF-β also modulates the functions of various stromal cells in the tumor microenvironment through miRNA-mediated gene regulation. TGF-β induces miR-494 in myeloid-derived suppressor cells (MDSCs), with this miRNA required for the accumulation and function of tumor-expanded MDSCs through PTEN suppression [129]. Inhibition of miR-494 reversed the ability of MDSCs and suppressed tumor growth and metastasis. TGF-β also promotes the induction of MDSCs by upregulating miR-21 and miR-155 [130]. Additionally, TGF-β reduces the tumor cytolysis activity of natural killer (NK) cells and abrogates its perforin polarization [131]. This inhibition of NK cell function is mediated by the induction of miR-183 by TGF-β and suppression of its target gene DAP12, which is important for surface NK receptor stabilization and downstream signals. Furthermore, it has been suggested that TGF-β-related miRNAs modulate the TGF-β-induced formation of cancer-associated fibroblasts. TGF-β-inducible miR-21 targets Smad7 and potentiates TGF-β-induced cancer formation [132].

Taken together, these findings suggest that miRNAs contribute to the modulation of the tumor microenvironment and immune suppression by TGF-β signaling through multiple mechanisms, including non-cell-autonomous functions in cancer cells and proximal functions in tumor stromal cells.

## 11. Concluding Remarks

In this review, I summarized the relationships between TGF-β signaling and the miRNA pathway. Previous studies have produced considerable conceptual advances regarding: (1) the regulation of miRNA expression by TGF-β signaling; (2) the modulation of TGF-β signaling by miRNAs; (3) the miRNA-mediated regulation of cell state transitions, which include EMT and EndMT; and (4) the crosstalk between miRNA and TGF-β pathways in cancer.

Crosstalk between TGF-β signaling and the miRNA biogenesis machinery has identified the unique transcription-independent roles of R-Smads, which suggests intimate crosstalk between transcriptional regulation and RNA processing. In fact, a recent study demonstrated that Smad2/3 facilitate the conversion of adenosine to N^6^-methyladenosine (m^6^A) on target RNA through the recruitment of the m^6^A methyltransferase complex, expanding the transcription-independent roles of R-Smads [133].

Studies regarding EMT-related miRNAs have highlighted that miRNAs constitute intrinsic bistable molecular switches in conjunction with EMT-inducing transcription factors [98,108]. This may be critical for modulating the reversibility of cell plasticity and the variability in cell differentiation. Further investigation of these feedback modules may increase our understanding of the reversibility of EMT at the single-cell level; of the intra- and inter-tumor heterogeneity of cancer phenotypes [134]; and of recently reported distinct EMT transition states [135]. Moreover, this may also provide further insight into several open questions regarding the EMT in vivo and in clinical samples [136].

Finally, TGF-β-related miRNAs may serve as potential therapeutic targets in various diseases, while clinically suitable methods of delivering small RNAs to various tissues should be improved [137,138]. Further studies of transcriptional and post-transcriptional regulation will yield a more comprehensive view of the alterations of the gene regulatory network modulated by TGF-β signaling and provide a basis for the development of new diagnostic and therapeutic approaches, which can be combined with improvements in the analytical analyses of miRNA networks [139,140,141].

## Abbreviations

Ago	Argonaute
BMP	bone morphogenetic protein
co-Smad	common-partner Smad
ECM	extracellular matrix
EMT	epithelial–mesenchymal transition
EndMT	endothelial–mesenchymal transition
EndMT-SP	EndMT-associated secretory phenotype
GDF	growth and differentiation factor
HBV	hepatitis B virus
HCC	hepatocellular carcinoma
HITS-CLIP	high-throughput sequencing after cross-linked immunoprecipitation
I-Smad	inhibitory Smad
lncRNA	long non-coding RNA
MALAT1	metastasis-associated lung adenocarcinoma transcript 1
MAPK	mitogen-activated protein kinase
MDCK	Madin Darby canine kidney
MDSC	myeloid-derived suppressor cell
MET	mesenchymal–epithelial transition
MMP	matrix metalloproteinase
NK	natural killer
NMuMG	normal murine mammary gland
PACT	protein activator of the interferon-induced protein kinase
PI3K	phosphatidylinositol 3 kinase
pri-miRNA	primary miRNA
RBP	RNA binding protein
R-Smad	receptor-regulated Smad
SBE	Smad binding element
TAM	tumor-associated macrophages
TβR	TGF-β receptor
TGF-β	transforming growth factor-β
TRBP	HIV transactivation response element (TAR)-binding protein
3′-UTR	3′ untranslated region
